# Ruxolitinib therapy for myelofibrosis in Austria: Consensus on therapy management

**DOI:** 10.1007/s00508-018-1365-5

**Published:** 2018-07-24

**Authors:** Maria-Theresa Krauth, Sonja Burgstaller, Veronika Buxhofer-Ausch, Günther Gastl, Klaus Geissler, Felix Keil, Peter Krippl, Thomas Melchardt, Andreas Petzer, Holger Rumpold, Thamer Sliwa, Stefan Wöhrer, Albert Wölfler, Heinz Gisslinger

**Affiliations:** 1 Department of Internal Medicine I, Division of Hematology and Hemostaseology, Medical University Vienna, Vienna, Austria; 2 Department of Internal Medicine IV, Klinikum Wels-Grieskirchen, Wels, Austria; 3 Department of Internal Medicine I, Hematology with Stem Cell Transplantation, Hemostaseology and Medical Oncology, Ordensklinikum Linz-Elisabethinen, Linz, Austria; 4 Department of Internal Medicine V, Division Hematology and Oncology, Medical University of Innsbruck, Innsbruck, Austria; 5 Fifth Medical Department, Hospital Hietzing, Vienna, Austria; 6 Third Medical Department, Hanusch Hospital, Vienna, Austria; 7 Department of Internal Medicine, LKH Fürstenfeld, Krankenhausverbund Feldbach, Fürstenfeld, Austria; 8 Third Medical Department, Division Hematology and Medical Oncology, Paracelsus Medical University Salzburg, Salzburg, Austria; 9 Internal Medicine II, Medical Oncology, Hematology, Gastroenterology and Rheumatology, Academic Teaching Hospital Feldkirch, Feldkirch, Austria; 10 Permedio Center for Personalized Medicine and Sanatorium Hera Vienna, Vienna, Austria; 11 Division of Hematology, Medical University of Graz, Graz, Austria

**Keywords:** Janus kinase 2, Myeloproliferative disorders, Primary myelofibrosis, Post-polycythemia vera myelofibrosis, Post-essential thrombocythemia myelofibrosis

## Abstract

The oral Janus associated kinase (JAK1/2) inhibitor ruxolitinib has been available for treatment of patients with intermediate or high-risk myelofibrosis in Europe since 2012. Since its introduction, the expertise of prescribing doctors with respect to ruxolitinib function, efficacy and adverse effects has consistently been augmented, resulting in therapy modalities that are better tailored to individual patients as well as in increased safety of the treatment. The present consensus on ruxolitinib therapy management has been elaborated by Austrian experts in myeloproliferative neoplasms in line with international treatment guidelines. Our recommendations aim to contribute to an improved management of patients with myelofibrosis treated with ruxolitinib.

## Introduction

Myelofibrosis (MF) is a Philadelphia chromosome (Ph)-negative myeloproliferative neoplasm (MPN) that can arise de novo (primary MF) or evolve from polycythemia vera (PV) or essential thrombocythemia (ET). It is characterized as a rare blood cancer, with an incidence of around 0.1–1 new cases per 100,000 Europeans per year [1].

The clinical picture of MF is characterized by extramedullary hematopoiesis with progressive splenomegaly, bone marrow fibrosis and cytopenia. Additionally, MF-related constitutional symptoms, such as night sweats, pruritus, profound fatigue, bone pain, weight loss, and cachexia occur that often severely compromise the quality of life (QoL) of patients [2]. Over the disease course, even more complications may arise, such as progressive hepatosplenomegaly, thromboembolic or bleeding events, or infections [3, 4].

The molecular mechanism behind MF is a dysregulation of the Janus associated kinase (JAK)/signal transducers and activators of transcription (JAK-STAT) signalling networks, which are necessary for cellular responses to cytokines and growth factors required for inflammation and normal hematopoiesis. Hyperactive signalling in JAK1/2 results both in malignant myeloproliferation as well as in the hyperinflammatory state that causes constitutional clinical symptoms [5, 6]. Consistent with overactivation of JAK/STAT signalling, MF is linked to the *JAK2 *V617F mutation in many patients [5].

Currently, allogeneic hematopoietic stem cell transplantation (allo-HSCT) is the only available therapeutic intervention with the potential to eliminate neoplastic stem cells and to cure patients with MF; however, only few patients are eligible for allo-HSCT given the need for appropriate donors as well as a high risk of treatment failure and treatment-related mortality [7]. In line with the dysregulated JAK/STAT signalling in MF, pharmacological targeting of the JAK pathway has become the preferred strategy for the treatment of patients with advanced or symptomatic MF. In Europe, the JAK1/2 inhibitor ruxolitinib is licensed for the treatment of adult patients with primary myelofibrosis (PMF), post-PV MF (PPV-MF) or post-ET MF (PET-MF; [8]), as well as for patients with PV who are resistant or intolerant to hydroxyurea [9].

Recently, this group has provided recommendations for the general management of MF in Austria [10]. In this review, the opinions and experiences of physicians prescribing ruxolitinib for MF are summarized and may collectively serve as guidelines for Austrian practitioners facing treatment decisions.

## Efficacy of ruxolitinib

### General efficacy

The efficacy of ruxolitinib in primary and secondary MF was assessed in two phase III clinical Controlled Myelofibrosis Study with Oral JAK Inhibitor Treatment (COMFORT) trials in which ruxolitinib action was demonstrated against placebo (COMFORT-I; [11]) or best applicable therapy (BAT, COMFORT-II; [12]). In both studies, reduction of spleen size was the primary endpoint, defined, in COMFORT-I, as a ≥35% reduction in spleen size after 24 weeks (reached by 41.9% in the ruxolitinib arm vs. 0.7% placebo; [11]) or as ≥35% reduction in spleen volume after 48 weeks in COMFORT-II (reached by 28% in the ruxolitinib arm vs. 0% BAT). In both studies, ruxolitinib improved MF-related symptom and QoL measures [11, 12]. The recent 5‑year follow-up analyses of both trials revealed that initial improvements in splenomegaly and symptom load were maintained with long-term therapy and are indicative of a slight survival advantage of ruxolitinib treated patients over those originally randomized to placebo or BAT [13–15].

### Disease-modifying effect

In the COMFORT trials, the survival benefit of ruxolitinib treated patients was apparent even when ruxolitinib treated groups were compared to control groups consisting largely of patients who had crossed over to ruxolitinib treatment early on, suggesting that early intervention might have the potential to improve disease outcome [15, 16]. A proposed mechanism by which ruxolitinib might have a life-prolonging effect is by reduction of systemic inflammation, which seems to be responsible for most constitutional symptoms and is strongly associated with a negative prognosis of MF [3, 17].

Ruxolitinib might act directly on the malignant bone marrow stem cell pool, resulting in reduced allele burden in patients carrying the *JAK2 *V617F mutation [15, 18]. In line with that, patients with the most pronounced decreases of allele burden after ruxolitinib therapy also exhibited the best spleen size responses [18].

The recent 5‑year update of COMFORT-II demonstrated that ruxolitinib might also slow down bone marrow fibrosis [15]. This was supported by data of patients who had received ruxolitinib in the phase I/II clinical trials: bone marrow fibrosis often stabilized or improved in the group that had been under ruxolitinib treatment for more than 5 years. In contrast, bone marrow fibrosis had worsened in patients within a historical comparator group who received BAT [19].

## Patient monitoring

### Diagnosis of MF and patient eligibility for ruxolitinib

#### Diagnosis

Diagnosis of PMF, as defined by the World Health Organization (WHO), is based on a combination of clinical, morphological, cytogenetic and molecular features (Table 1; [20]).

**Table 1:** World Health Organization (WHO) diagnostic criteria for PMF (Table adjusted from [20])

WHO diagnostic criteria for PMF. For meeting the requirement of PMF, all 3 major criteria, plus ≥2 minor criteria must be met
*I. Major criteria*
a. Megakaryocyte proliferation, including small-to-large megakaryocytes, with aberrant nuclear/cytoplasmic ratio and hyperchromatic and irregularly folded nuclei and dense clustering accompanied by either reticulin and/or collagen fibrosis or in the absence of reticulin fibrosis (i.e., prefibrotic PMF), the megakaryocyte changes must be accompanied by increased marrow cellularity, granulocytic proliferation, and often decreased erythropoiesis
b. Not meeting WHO criteria for chronic myelogenous leukemia, polycythemia vera, myelodysplastic syndromes, or other myeloid neoplasm
c. Demonstration of *JAK2 *V617F or other clonal marker or no evidence of reactive marrow fibrosis
*II. Minor criteria*
a. Leukoerythroblastosis
b. Increased serum lactate dehydrogenase
c. Anemia
d. Palpable splenomegaly

*PMF* primary myelofibrosis, *WHO* World Health Organization

The individual risk of patients with PMF can be estimated using specific scoring systems, such as the International Prognostic Scoring System (IPSS; [3]), a classification based on the prognostic impact of age and distinct clinical characteristics at the patient’s first presentation (Table 2). Recently, more refined systems have been developed, such as the dynamic IPSS score (DIPSS) that allows prognostication at any time during the clinical course, and its derivative DIPSS plus that takes additional risk factors into account, including unfavorable karyotype, platelet count, and transfusion dependency (Table 2; [3, 4, 21]). Using these models, patients are assigned to risk categories low, intermediate-1, intermediate-2 and high that differ with respect to their survival probability.

**Table 2:** Risk stratification of patients with myelofibrosis (MF) according to the International prognostic scoring system (IPSS; [3]), dynamic IPSS (DIPSS), [4], and DIPSS plus [21]. (Table adjusted from [27])

Risk category	Scale	Estimated survival (years)
*IPSS*	*No. of risk factors* ^*a*^	*Median (95% CI)*
Low	0	11.3 (9.8–15.1)
Intermediate-1	1	7.9 (6.6–9.5)
Intermediate-2	2	4.0 (3.6–4.9)
High	≥3	2.3 (1.9–2.6)
*DIPSS*	*Prognostic score* ^*b*^	*Median*
Low	0	NR
Intermediate-1	1 or 2	14.2
Intermediate-2	3 or 4	4.0
High	5 or 6	1.5
*DIPSS plus*	*Prognostic score* ^*c*^	*Median*
Low	0	15.4
Intermediate-1	1	6.5
Intermediate-2	2 or 3	2.9
High	4–6	1.3

*NR* not reached

^a^Risk factors include age >65 years, constitutional symptoms (defined as weight loss >10% of baseline value in the year preceding diagnosis and/or unexplained fever or excessive sweats persisting for more than 1 month), hemoglobin <10 g/dl, white blood cell count >25 × 10^9^/l, and peripheral blood blasts ≥1%

^b^Risk factors include age >65 years (1 point), constitutional symptoms (1 point), hemoglobin <10 g/dl (2 points), white blood cell count >25 × 10^9^/l (1 point), and peripheral blood blasts ≥1% (1 point)

^c^Scoring is based on DIPSS risk categories (low risk, 0 points; intermediate-1 risk, 1 point; intermediate-2 risk, 2 points; high risk, 3 points) and additional risk factors (unfavorable karyotype, 1 point; platelet count <100 × 10^9^/l, 1 point; transfusion need, 1 point)

#### Eligibility for ruxolitinib treatment

The European LeukemiaNet (ELN) and the Italian Association for Hematology (SIE) have recently issued evidence-based criteria for the eligibility of MF patients for ruxolitinib therapy. According to these guidelines, ruxolitinib is strongly recommended for patients with intermediate-2 or high-risk disease according to IPSS. Additionally, a weak recommendation was issued for intermediate-1 patients with symptomatic or severe splenomegaly (≥15 cm below costal margin). Moreover, the ELN-SIE association compared the potential of several assessment methods to select patients for ruxolitinib therapy according to QoL. Eventually, the MPN10 score was recommended, a brief disease-specific tool available in multiple languages that was longitudinally applied in the COMFORT-I trial [22]. A MF symptom/QoL-based recommendation for ruxolitinib was issued for patients with an MPN10 score >44, refractory severe pruritus, unintentional weight loss (>10% within the last 6 months) or unexplained fever regardless of the DIPSS score. In addition, patients with increased thromboembolic risk should be considered for ruxolitinib treatment [22].

#### Early onset treatment with ruxolitinib

The ELN-SIE association discouraged early onset use of ruxolitinib for MF due to a lack of direct evidence [22]. It is still felt that ruxolitinib treatment should be discussed as an option for patients with less progressed disease who may benefit of an early QoL improvement and disease-modifying effect. In an observational study, a shorter time window between diagnosis and treatment onset, as well as less progressed splenomegaly (<10 cm below costal margin) or bone marrow fibrosis (<grade 3) correlated with better responses to ruxolitinib [23].

While the registration trials were limited to intermediate-2 and high-risk patients, the phase II study ROBUST and the phase IIIb study JUMP additionally included patients with intermediate-1 risk MF. Both showed that patients of intermediate-1 risk treated with ruxolitinib achieved comparable reductions of spleen volume and constitutional MF symptoms as patients in intermediate-2 and high-risk MF groups [24–26]. In addition, the safety profile in the intermediate-1 risk subgroup did not differ from other risk categories and was comparable to what was reported in the COMFORT studies [11, 12, 27]. A recent update of the JUMP study demonstrated improved spleen size reduction and constitutional symptom improvement on ruxolitinib treatment in patients with intermediate-1 MF compared to higher risk groups after 72 weeks of treatment. Additionally, lower-risk patients remained on treatment longer than high-risk patients, and fewer patients discontinued ruxolitinib therapy because of adverse effects [25].

##### Consensus

The evidence-based criteria recently issued by the ELN-SIE consortium can help in the decision of whether to prescribe ruxolitinib for MF. Following current European guidelines, ruxolitinib is recommended for treatment of patients with primary, post-ET or post-PV MF of intermediate-2 and high risk according to IPSS/DIPSS scoring. Additionally, ruxolitinib therapy is weakly indicated for patients with intermediate-1 risk but with symptomatic or severe splenomegaly. Furthermore, patients with high disease burden from constitutional symptoms should be considered for ruxolitinib treatment regardless of the IPSS or DIPSS score. Therefore, systematic and quantitative assessment of MF-associated symptoms with tools, such as the MPN10 score is recommended prior to taking treatment decisions. Patients with an MPN10 score ≥44 should be considered for ruxolitinib treatment [22].

### Precautions before treatment

#### Renal function

In volunteers with intact renal function or with varying degrees of renal impairment, plasma retention of ruxolitinib metabolites increased proportionally to the severity of renal impairment [28]. Since the effect of increased metabolite exposure is unknown, renal function should be assessed routinely prior to onset of ruxolitinib treatment. Patients with a baseline creatinine clearance of less than 30 ml/min should receive reduced starting doses of ruxolitinib [29]. Renal function should be routinely monitored, and dose escalations should be made stepwise and slowly. Patients diagnosed with severe renal impairment while under ruxolitinib therapy should be especially carefully monitored, and dose modifications should be applied with respect to safety and efficacy [29].

#### Hepatic function

Patients with mild to severe hepatic impairment displayed longer ruxolitinib retention times compared to healthy subjects [28]. Consequently, patients exhibiting any degree of hepatic impairment (Aspartate aminotransferase [AST]/Alanine aminotransferase [ALT] >2.5 × upper limit of normal [ULN], total bilirubin >1.5 × ULN) should receive reduced doses of ruxolitinib, and the dosage should be increased stepwise and slowly (see dosing; [29]). Furthermore, a patient’s coagulation status should be regularly monitored.

#### Hematopoietic function

Patients with platelet counts ≥50 × 10^9^/l and absolute neutrophil count (ANC) of ≥1.0 × 10^9^/l should be considered for normal ruxolitinib dosage [29]. For dose modifications according to hematopoietic parameters see section “dosing”.

#### Infections

Due to the increased vulnerability for infections, patients should be assessed for the risk of developing severe bacterial, mycobacterial, fungal, and viral infections prior to prescription of ruxolitinib. Pre-existing infections should be excluded by lung radiography, urine analysis, blood analysis (albumin, total protein, triglyceride and cholesterol, QuantiFERON® test [Cellestis GmbH, Germany]) and serology for human immunodeficiency virus (HIV), hepatitis, cytomegalovirus (CMV), Epstein-Barr virus (EBV) and herpes zoster. In patients with increased risk of viral infections (such as pre-existing hepatitis B infection) adequate antiviral therapy (e. g., tenofovir) should be considered. Acute infections with herpes zoster should be treated with e. g. valaciclovir 500 mg daily until 1 month after termination of the infection. For patients with recurrent herpes zoster infections, prophylactic valaciclovir is recommended.

##### Consensus

Before initiation of ruxolitinib treatment, renal and hepatic function should be assessed and then monitored throughout the course of disease because dose modifications may be required. Physicians should be aware of the increased risk of infections and screen for hepatitis, herpes and tuberculosis before treatment onset.

### Monitoring of treatment response

#### Spleen size

Spleen size is currently the most frequently used prognostic indicator of MF. In a pooled analysis of the COMFORT cohorts, increased baseline spleen volume at treatment onset correlated with reduced survival [30]. Accordingly, spleen size is also a sensitive marker for ruxolitinib response. In both COMFORT trials, patients who achieved higher spleen size reductions displayed better overall survival [23]. We recommend determining splenomegaly, at the minimum at treatment onset, by volumetry via magnetic resonance imaging (MRI) or computed tomography (CT), since imaging-based detection of spleen volume could better predict leukemia-free and overall survival than physical examination [31].

#### Symptom control

The reduction of constitutional symptoms such as night sweats is another useful parameter to assess therapy response. We recommend carrying out a complete disease assessment for every patient before treatment onset, and documenting MF-related symptoms with tools such as MPN10 or the Myeloproliferative Neoplasm Symptom Score (MPN-SAF TSS), to facilitate documentation of treatment response and estimation of disease prognosis [32].

#### Lactate dehydrogenase

Typically, elevated serum levels of lactate dehydrogenase (LDH) accompany PMF, which might reflect tumor killing or hemolysis [33]. Accordingly, a decrease in LDH levels may correlate with treatment response [34].

##### Consensus

Treatment response should be monitored with respect to spleen size and blood counts. A reduction of ≥25% of the baseline spleen size should be considered as a satisfactory response. Additionally, constitutional symptom assessment (especially night sweat reduction) can help to monitor treatment response. In the absence of a clinically meaningful response to ruxolitinib 6 months after its initiation, treatment should be discontinued.

## Treatment regimens

### Dosing

#### Starting dose

Following the experiences of the COMFORT trials, dosing recommendations are based on platelet counts. European guidelines correspond to the scheme of COMFORT-II: for patients with a baseline platelet count between 100–200 × 10^9^/l, a starting dose of 15 mg twice daily (bis in die, bid), and for those with levels of >200 × 10^9^/l, 20 mg bid are recommended [29].

In clinical practice, also lower treatment doses may lead to significant responses. In line with that, dose reductions often did not significantly interfere with ruxolitinib efficacy. In COMFORT-I, lowering of the therapeutic dose to final titrated doses of ≥10 mg bid still led to clinically significant reductions in spleen volume and improvement in MF-related symptoms [35, 36]. Ruxolitinib efficacy seems to decline significantly only at final titrated doses ≤10 mg bid, with a reduced response of spleen size and constitutional symptoms [23].

#### Reduced dosing regimen for patients with thrombocytopenia and neutropenia

Approximately one quarter of MF patients display baseline platelet counts of less than 100 × 10^9^/l as a consequence of their disease [37]. Ruxolitinib should only be prescribed for patients with a baseline platelet count of more than 50 × 10^9^/l [29]. In two small studies it was found that a clear therapeutic benefit can be obtained in patients with baseline platelet counts of 50–100 × 10^9^/l employing a careful regimen based on starting doses of 5–10 mg bid. In these patients, ruxolitinib was also generally well tolerated [37, 38].

Neutrophil counts should be assessed before and regularly after ruxolitinib treatment onset; if they decline below 0.5 × 10^9^/l, treatment should be interrupted. After a recovery to 0.75 × 10^9^/l, treatment should be restarted with concomitant dose reduction [29].

#### Reduced dosing regimen for impaired hepatic or renal function

Analysis of the pharmacokinetic and pharmacodynamic data for ruxolitinib led to the recommendation that patients with platelet counts of 100–150 × 10^9^/l and any degree of hepatic impairment, or with moderate or severe renal impairment should be treated with reduced starting doses [29]. For patients with severe renal disease receiving dialysis, ruxolitinib administration should be coordinated with the dialysis scheme [29]. Ruxolitinib should not be administered in patients with end-stage renal disease not requiring dialysis, or with moderate or severe renal impairment or hepatic impairment with concomitant platelet counts of less than 100 × 10^9^/l [29].

##### Consensus

We suggest a bottom-up strategy, starting at 5 or 10 mg bid for patients with normal platelet counts. This strategy is preferable to a top-down scheme, in which high doses may lead to toxicities potentially necessitating dose adjustments or temporary discontinuation. In our experience, long-term compliance with ruxolitinib treatment was better in patients treated with a bottom-up dosing scheme. Blood counts should be carefully monitored in the beginning and dose modifications should be based on platelet and neutrophil counts alongside the clinical response. If no reduction of the spleen volume is observed within the first 4 weeks, the dose should be carefully increased. We suggest a subsequent dose increase of 5 mg per day every week. For patients with renal or hepatic insufficiencies or reduced platelet counts, ruxolitinib should be initiated with 5 mg daily, and the dosage should be retained at low levels (5 mg bid) or carefully escalated. Similar to patients with reduced platelet counts, caution is required for patients receiving therapeutic anticoagulation due to the increased risk of bleeding. For older patients, we recommend to start at 5 mg once or twice daily, and to increase the dosage according to blood counts.

### Treatment discontinuation

#### Ruxolitinib withdrawal syndrome

In a phase I dose escalation study 5 out of 47 patients (11%) who had discontinued ruxolitinib treatment experienced withdrawal complications requiring hospitalization [39]. Especially sudden ruxolitinib discontinuation may lead to serious adverse events with symptoms reminiscent of a systemic inflammatory response syndrome (SIRS): examples are severe symptomatic splenomegaly, fever and acute hemodynamic decompensation [40]. Patients often appear somnolent, and imaging of the lungs may reveal cloudy shadows, potentially due to inflammatory exudates, that are of non-autoimmune origin. Upon reinstallation of therapy, the condition of patients usually improves rapidly, and vital parameters stabilize. Systemic inflammation can often be ameliorated with concomitant cortisol treatment.

The MF symptom scores were reported to return to baseline values within 7 days after dose discontinuation [11], which is the basis for the recommendation for a gradual tapering in cases where ruxolitinib needs to be discontinued [29]. In cases of autonomous discontinuation, the cause for the acute condition might be overlooked and misdiagnosed as pneumonia or ruxolitinib-induced immune complications. To prevent sudden discontinuation in situations where the patient is unable to inform treating doctors of the ruxolitinib treatment (as when a patient is hospitalized following an accident), issuing of a medical ID might be useful. Especially intensive care patients are at an increased risk to develop withdrawal symptoms following ruxolitinib discontinuation, which might be misinterpreted for other clinical conditions such as refractory septic shock [39]. Since ruxolitinib is often applied as a bridging therapy before allo-HSCT, it is imperative to continue ruxolitinib until conditioning chemotherapy, to prevent withdrawal syndromes in the context of transplantation.

##### Consensus

We generally recommend ruxolitinib as a permanent therapy, and discontinuation should be considered only in exceptional cases, such as in the case of ruxolitinib ineffectiveness or very severe adverse effects. Preferably, ruxolitinib therapy should be continued with adjusted dosages. In our experience, a clinical benefit can be obtained even with moderate doses, and even patients that lack measurable spleen size reductions reported ameliorated MF-related symptoms and increased subjective well-being. Gradual tapering of the medication over a long duration is mostly unproblematic but should occur only under close supervision.

## Ruxolitinib toxicity

### Hematologic toxicity

Since JAK2 plays essential roles in the transduction of signals from erythropoietin and thrombopoietin receptors, cytopenia is a frequent and dose-dependent side effect of ruxolitinib therapy [6]; however, the experiences from both COMFORT studies showed that cytopenia rarely requires treatment discontinuation and can most often be effectively managed with dose modifications, temporary treatment interruptions, as well as red blood cell (RBC) transfusions in the case of anemia [6, 11, 12].

#### Anemia

Anemia is a frequent consequence of MF. The additional inhibition of erythropoiesis through ruxolitinib leads to a further decrease in hemoglobin values during the first weeks of therapy, which usually recover and subsequently stabilize as a consequence of the ruxolitinib response [11, 12]. Discontinuation due to anemia was very infrequent in clinical studies [11, 12].

#### Thrombocytopenia

Thrombocytopenia is an independently weighted risk factor in the DIPSS plus score and correlates with decreased survival probability for MF patients [21]. In the COMFORT-I study, 56% of patients required dose reductions or treatment interruptions due to thrombocytopenia; however, most of them resulted in stabilization of mean platelet counts after the first 8–12 weeks of treatment (a time point at which decreases in platelet counts primarily occurred; [6, 35]). In the COMFORT trials, grade 3/4 episodes of bleeding were uncommon with ruxolitinib and occurred at rates similar to those with placebo, suggesting that treatment interruption and dose management effectively counteracted ruxolitinib-induced thrombocytopenia [11, 12].

#### Neutropenia

Due to the neutropenia it may induce, neutrophil counts should be closely monitored before and during ruxolitinib therapy. Patients with neutrophil counts over 0.5 × 10^9^/l are eligible for ruxolitinib treatment, and values below are a reason for treatment interruption. Patients with a neutrophil count of 0.5–1.0 × 10^9^/l should receive decreased doses of ruxolitinib.

##### Consensus

Clinicians should note that when initiating ruxolitinib therapy, hemoglobin values may decrease. Upon diagnosis of anemia, ruxolitinib-independent causes such as iron-, folic acid- and vitamin B12 insufficiencies as well as hemolysis should be excluded. At a hemoglobin threshold of 8 g/dl, transfusions with erythrocyte concentrates can be considered to achieve stabilization. Due to the increased symptom load of cardiovascular patients, they should receive transfusions already at 9 g/dl. To prevent iron overload, iron chelation therapy with deferasirox may be additionally applied. Furthermore, folic acid and a single shot therapy with steroids may improve hematopoiesis; it is a matter of debate whether erythropoiesis-stimulating agents (ESA) are useful for treatment of ruxolitinib-induced anemia, as erythropoietin receptor signals require functional JAK signalling; however, in a recent retrospective study it was shown that application of ESAs could improve anemia in ruxolitinib treated patients [41].

Careful and appropriate monitoring of hematologic parameters during treatment initiation is imperative, and dose adjustments should be tailored to the clinical response and hematologic side effects of each patient. Ruxolitinib dosage should be adjusted according to platelet counts described in the manufacturer’s instructions and increased stepwise during course of treatment. Platelet transfusions can assist to bridge critical phases; however, we do not recommend thrombopoietin receptor agonists. Larger surgical procedures can be performed at a platelet count of ≥100 × 10^9^/l.

### Nonhematologic toxicity

In the COMFORT trials, reported nonhematologic toxicities were rare and mostly mild [11, 12].

#### Infections

The most important nonhematologic risk factor under ruxolitinib treatment is the increased vulnerability to bacterial infections due to neutropenia. Thereby, urinary tract and herpes zoster infections were reported in patients in the ruxolitinib arm of the COMFORT studies [13, 15]. More rarely, pneumonia, sepsis and tuberculosis infections were also observed [15]. The recent 5‑year follow-ups of the COMFORT studies confirmed these observations; however, the incidence of infections did not increase in the long term [15, 16]. Other studies described the occurrence of bilateral toxoplasmosis retinitis, reactivation of hepatitis B, and reactivation and dissemination of tuberculosis [42–44].

#### Secondary malignancies

As for other immunomodulatory agents, ruxolitinib might predispose to an increased risk for secondary malignancies [29]. Patients occasionally reported skin cancers including basal cell, squamous cell, and Merkel cell carcinomas [45]. Therefore, it is recommended that the skin should be regularly examined while patients are treated with ruxolitinib [29].

Recently, in one reference center in Austria, diffuse large B‑cell lymphomas evolved in 4/69 patients (5.8%) on JAK1/2 inhibition compared to 2/557 (0.36%) with conventional treatment. Lymphomas occurring during ruxolitinib treatment were preceded by a pre-existing B‑cell clone in 3 patients tested and arose around 1–3 years after treatment onset; however, all patients were in an advanced disease stage, required transfusions and were classified as high risk according to IPSS. In addition, some of them were massively pretreated with other cytoreductive agents such as hydroxyurea or pipobroman, sometimes over several years [46].

#### Other adverse effects

Another frequent side effect is pain in the bone or the splenic capsule, which accompanies the ruxolitinib-induced remodelling processes in the bone marrow and spleen. A potential weight gain is often beneficial for MF patients that carry a certain cachexia risk. Additionally, weight gain correlates with treatment response [47] and was associated with a strong survival advantage [48]. Naturally, weight gain should stay within a healthy range.

##### Consensus

Due to a general vulnerability for infections, patients’ individual risk should be assessed prior to ruxolitinib therapy. Physicians should carefully observe patients prior to receiving ruxolitinib for signs and symptoms of infections and initiate appropriate and timely treatment.

To prevent the emergence of complicated infections, patients should be instructed to consult a doctor in case of fever. In addition, prophylactic antibiotic prescriptions or keeping medication at home should be considered for emergency situations. In the case of repetitive pneumonia, continuous application of antibiotics might be helpful.

Reduction of disease burden subsequent to ruxolitinib treatment is sometimes associated with weight gain. A concomitant increase in cholesterol values should be a reason for concern, as it might be indicative of increased cardiovascular risk. A potential risk for secondary malignancies under ruxolitinib treatment should be considered. As more data arise on this topic, we think that screening for pre-existing lymphoproliferative diseases (especially B‑cell clonality) should also be considered before starting ruxolitinib therapy. For example, testing for clonal immunoglobulin rearrangements via qualitative PCR may be useful.

## Co-medication under ruxolitinib

### Interactions with substances metabolized via CYP3A4

MF occurs mostly in the elderly population that frequently suffers from various comorbidities; therefore, patients often require medication for other indications concomitant to treatment of MF. Ruxolitinib is metabolized via the cytochrome P (CYP) family members CYP3A4 and, with lower efficiency, CYP2C9. These enzymes are active in the kidney and the liver and are potently inhibited by a few classes of pharmacological compounds, such as the antibiotics clarithromycin or telithromycin or the antifungals fluconazole and ketoconazole [29]. A more detailed list of CYP3A4 and CYP2C9 inhibitors can be found in the prescribing information and in Table 3. Co-application of these substances may lead to an increased plasma half-life of ruxolitinib. Therefore, in a setting where concomitant administration of ruxolitinib and a CYP3A inhibitor is required, ruxolitinib should be applied in a reduced dosing scheme [29]. For example: in patients taking ruxolitinib, the dose of daily applied fluconazole should not exceed 200 mg [29]. Concomitant use of strong CYP3A4 inhibitors and ruxolitinib should be accompanied by increased monitoring of clinical ruxolitinib response and hematologic parameters ([29]; Table 3).

**Table 3:** Examples of substances that strongly or moderately inhibit or enhance CYP3A4. (Adapted from [29, 56])

Substance class	Substance	Interaction with ruxolitinib
Strong CYP3A4 inhibitors	Clarithromycin, telithromycin (antibiotic)	Enhancing ruxolitinib action (requires ruxolitinib dose reduction)
	Nefazodone (antidepressant)	
	Itraconazole, ketoconazole, voriconazole, posaconazole (antifungal)	
	Boceprevir, ritonavir, indinavir, saquinavir, nelfinavir, amprenavir, lopinavir, telaprevir (virus protease inhibitors)	
	Conivaptan (vasopressin inhibitor)	
Moderate CYP3A4 inhibitors	Erythromycin, fluconazole, aprepitant, verapamil, diltiazem, grapefruit, grapefruit juice	Enhancing (Monitor cytopenias, consider dose reduction)
CYP34A inducers	Rifampicin, carbamazepine, phenobarbital, St. John’s wort	Reducing ruxolitinib action (Consider ruxolitinib dose increase)

### Anticoagulation

Because ruxolitinib can induce or worsen thrombocytopenia, concomitant anticoagulation (such as oral anticoagulants, or acetylsalicylic acid) may increase patients’ risk for bleedings. We suggest careful monitoring of platelets and coagulation parameters as well as awareness of clinical signs of bleeding.

## Future directions

### Restoration of ruxolitinib response after brief withdrawal

A recent case study suggested that patients might regain sensitivity to ruxolitinib following temporary treatment discontinuation. In two patients with ruxolitinib resistance, ruxolitinib was discontinued by gradual dose tapering. Subsequently, ruxolitinib was reintroduced, and in both patients, the initial responsiveness to the drug was partially restored [34].

### Combination of ruxolitinib with cytoreductive therapy

Emerging preclinical and clinical data suggest that combination of ruxolitinib with an anti-inflammatory or cytoreductive partner might improve its efficacy and ability to reverse disease [49, 50]. For instance, a high proportion of patients responded to a combination of ruxolitinib and the hypomethylating agent azacitidine (48% achieved a >50% spleen length reduction at 24 weeks; [51]). Case studies demonstrated effective combinatorial schemes with hydroxyurea [52] and interferon alpha-2a [53] in patients with severe PMF or PV. Combined therapy of ruxolitinib with interferon may be particularly beneficial: it targets both malignant stem cells and inflammation, breaking the vicious cycle of an inflammatory environment that enhances the oncogenic milieu (which fuels inflammation in return; [54, 55]).

## Conclusion

Ruxolitinib is a relatively safe and effective treatment option for patients with MF. Nevertheless, patients should be carefully selected with respect to potential risks and benefits of ruxolitinib therapy. Therefore, disease stage and burden as well as hematological, renal and hepatic parameters should be evaluated pre-treatment, and, in patients eligible for ruxolitinib, continuously over the disease course. Both clinical hematologists and patients should be aware of the danger of sudden ruxolitinib withdrawal that may result in severe systemic inflammatory symptoms. In the future, we expect that ruxolitinib will be combined with anti-inflammatory or cytoreductive agents to increase its efficacy and disease-modifying effect.
